# Influence of self-efficacy on male military pilots’ capability to handle special situations: a moderated mediation model

**DOI:** 10.1038/s41598-023-38009-9

**Published:** 2023-07-04

**Authors:** Rui Qiu, Yue Gong, Yang Cao, Xuqun You, Xia Zhu

**Affiliations:** 1 Department of Military Medical Psychology, Air Force Medical University, Xi’an, 710032 China; 2 School of Psychology, Shaanxi Normal University, Xi’an, 710032 China

**Keywords:** Psychology, Health care

## Abstract

This study aimed to explore the mediating and moderating effects of resilience and perseverance on pilots’ self-efficacy and capability of handling special situations. Using cluster sampling, 251 pilots’ self-efficacy, special flight situation handling capability, resilience and perseverance were assessed using standardized scales. Pilots with high self-efficacy can improve their resilience to enhance their capability to handle special situations. An analysis was performed that included perseverance in the mediation model, and results showed that the influence of self-efficacy on special situation handling through resilience was moderated by perseverance. The relations between self-efficacy and special flight situation handling capability present a moderated mediation model. A pilot’s capability of handling special situations, ensuring flight safety and combat capability may be enhanced by improving their self-efficacy, resilience, and perseverance.

## Introduction

Special flight situations refer to a series of special situations that occur during a flight. Improper handling of special flight situations can result in serious consequences such as air crashes^1^. For example, on March 8, 2014, MH370 went missing; on March 21, 2022, MU5735 crashed; and Chengdu J-7/F-7 Fighter crashed^2–4^ on June 9, 2022. In civil aviation, special flight circumstances typically involve the safety of the fuselage, systems, and extreme weather phenomena, as well as human error. In contrast, in military aviation there are two primary categories of special flight circumstances: those encountered during regular training and those encountered during mission execution. The former is similar to civil aviation, whereas the latter involves various factors, such as operational errors resulting from the pilot’s combat stress reactions, damage to aircraft parts caused by attacks, and blind flying due to the inability to communicate with ground control. Therefore, the identification of special situations during flight in a timely manner is urgently needed to properly handle them. Many studies have been undertaken by numerous researchers on mechanical control systems and other aspects of flight. For example, Chen et al., constructed the STAMPHFACS analysis framework to identify risk factors for special events that negatively impact flight safety, while Liu et al., constructed a risk profile model for bird strikes in airports through the Artificial Neural Network (ANN). Otherwise, Pan et al., applied Structural Equation Modeling (SEM) to analyze safety risks and action mechanisms in the flight area of civil airports, and Yan et al., applied the Interpretation Structure Model (ISM) to explore and analyze risk factors that influence the safety of regional control operations^5–8^. Most of these analyses and investigations are applicable to engineering and control systems. However, not many analyses have examined psychological factors of pilots while flying, and not many analyses and constructions of psychological models for special flight situations have been undertaken. The study of flight special circumstances and their influencing factors in aviation psychology is still relatively new. In this study, we define the ability to handle flight special circumstances as a pilot’s capacity to accurately receive malfunction information during special situations, and make appropriate decisions by carefully considering various factors. Therefore, this study introduces self-efficacy, resilience and perseverance into the model of factors that potentially influence an individual’s special situation handling capability and explores the influence of an individual’s psychological make-up on his/her capability of handling special situations.

### Relations between self-efficacy and special situation handling capability

Special situations refer to all kinds of special cases that occur during a flight. The sound capability of handling special situations are a prerequisite for ensuring flight safety. In 1977, Albert Bandura put forward the definition of self-efficacy, which refers to an individual’s belief of achieving behaviors and goals in a special field^9^. An individual with high self-efficacy will face difficult situations with a positive attitude, make full use of his abilities to achieve a goal, and promote the development of competence^10,11^. Therefore, pilots who possess high self-efficacy are able to trust their own capabilities, effectively allocate personal and crew resources, and navigate challenging in-flight scenarios proficiently. When handling special situations, a pilot needs to be able to identify problems in a timely manner, accurately judge the situation, and correctly handle them. The capability to manage special situations calmly requires not only excellent flight skills, but also sound psychological well-being and self-efficacy. One should be confident in his/her decision-making and handling capability to ensure flight safety^12,13^.

### The mediating effect of resilience between self-efficacy and special situation handling capability

Resilience, an individual’s instinctive response based on the defense-response mechanism, can be defined as an individual’s ability to quickly mobilize his/her capabilities and resources to make adjustments when facing a major crisis, and to cope with and successfully overcome difficulties^14^. Masten et al., put forward the indirect factor model of resilience, and according to this model, high self-efficacy, as a protective factor, first plays a positive role in resilience, and resilience, an intermediary factor, plays a positive role in the developmental outcome of special situation handling capability. Low self-efficacy, as a risk factor, will eventually enable an individual to attain sound capability of handling special situations due to the retardation effect of resilience^15^.

### The moderating role of perseverance between self-efficacy and resilience

Besides the direct and indirect effects of self-efficacy and resilience on special situation handling, perseverance may also play a moderating role between self-efficacy and resilience, thus influencing an individual’s capability of handling special situations. Duckworth et al. put forward the concept of perseverance in 2007, which refers to the persistence and the will to work diligently and unremittingly towards a goal^16^. Perseverance involves two aspects: one is persistence in the face of difficulties, and the other is enthusiasm for struggle. Accordingly, we assume that an individual with high perseverance is more capable of resisting adversity than an individual with low perseverance^17^. Based on the 3C (commitment, control and challenge) structure of perseverance, an individual with strong perseverance can bring more positive meaning to life, take more active actions to solve a crisis, and regard change as a means of growth^18^. Therefore, perseverance may play a moderating role between an individual’s self-efficacy and resilience through the above three dimensions (commitment/control/challenge).

## Methods

### Measuring object

Two-hundred and fifty-one members of the flight brigade in H City were selected using cluster sampling. With the district team as a unit, a standard group test was administered to participants by trained psychology postgraduates. In the testing process, the following procedures and requirements were strictly followed: less than 30 respondents were tested at any one time; the test instructions were standardized; unified questionnaires were administered in dedicated testing rooms. The purpose was to collect data quickly and accurately. All methods were carried out according to the experimental guidelines of Air Force Military Medical University, approved by the Ethics Review Committee of the Department of Military Medical Psychology, and all subjects signed informed consent forms.

To ensure the absolute security and reliability of pilots’ personal data, the research group used paper questionnaires for testing. Which consists of test for measuring the Self-Efficacy, special flight situation handling capability, Resilience and Grit of the subjects. After missing data were eliminated, data for 249 respondents were retained. After data cleaning, 224 respondents were retained for analysis, yielding an effective response rate of 89.96%. All respondents were male, with an average age of 21.99 ± 0.925, and an average flight duration of 175.81 ± 104.403 h.

### Measuring tools

#### Self-efficacy scale

Schwarzer et al. developed a self-efficacy scale consisting of ten items^19^, that are answered using a 5-point Likert scale (0 = “completely disagree”; 4 = “completely agree”) with higher scores indicative of greater self-efficacy. Ji et al.^20^ used the Chinese version of this scale to assess self-efficacy in civil aviation pilot cadets and found that self-efficacy influenced their capability to judge situations. In this scale, Cronbach α = 0.934; construct validity *χ*^*2*^*/df* = 3.009; RMSEA = 0.095; CFI = 0.960; TLI = 0.946; and SRMR = 0.035.

#### Test of special flight situation handling capability

The pilots’ capability to judge the situation was investigated using the situational judgment technique developed by Hunter et al.^21^. The technique was translated into a Chinese version and revised to reflect actual tasks and situations undertaken by Chinese pilots. Finally, a test of special flight situation handling capability, consisting of three parts (emergency response, decision judgment, and special situation), fifteen scenarios, and 90 items, was determined. The study involved 15 special situations, with six topics measured for each situation. The first topic focused on the participant’s ability to identify special situations (“what have you found”), while the second topic measured their diagnostic ability in identifying the causes of the current situation (“what causes the current situation”). The third topic evaluated the participant’s ability to handle the situation by choosing a corresponding strategy (“which corresponding strategy can be chosen for the problems in this scene”). Topic four assessed the participant’s risk assessment ability by identifying the advantages and disadvantages of different disposal strategies (“what are the advantages or disadvantages of different disposal strategies”), while topic five examined the influential factors of the participant’s background in their decision-making process (“what factors may be affected when choosing the disposal strategy”). Finally, topic six measured the participant’s ability to make a final decision (“final choices and decisions”). For each topic, three options were provided: a score of 0 represented a “typical wrong choice”, a score of 2 indicated the “best choice”, and a score of 1 was given for a choice that was between the two (“typical wrong choice” and “best choice”).During the analysis, the total score of the test scale for special flight situation handling capability was calculated. The higher the total score, the stronger the respondents’ special flight situation handling capability.

#### Connor Davidson Resilience Scale

Conner et al., developed the Connor Davidson Resilience Scale^14^ which was subsequently translated into a Chinese version and revised^22^. The scale consists of three dimensions—tenacity, strength and optimism and includes 25 items, answered using a 5-point Likert scale. In this scale, Cronbach α = 0.933; construct validity *χ*^*2*^*/df* = 2.681; RMSEA = 0.087; CFI = 0.928; TLI = 0.904; and SRMR = 0.050.

#### Grit Scale

Duckworth et al., put forward and developed the Grit Scale^23^ which was revised and translated into Chinese by Zhang et al. They subsequently tested it with military college students and results showed good reliability and validity^24^. This scale consists of two dimensions (interest and persistence), includes 12 items and is answered using a 5-point Likert scale (0 = “completely consistent”; 1 = “a bit consistent”; 2 = “partly consistent”; 3 = “mostly consistent”; 4 = “completely consistent”). In this scale, Cronbach α = 0.808; construct validity *χ*^*2*^*/df* = 2.641; RMSEA = 0.086; CFI = 0.931; TLI = 0.897; and SRMR = 0.078.

### Data analysis

SPSS28.0 and Mplus8.3 were used to perform a common method deviation test, descriptive statistical analysis, correlation analysis, and analysis and tests of mediation and adjustment effects.

## Results

### Common method deviation test

The Harman single factor method was utilized to test the common method deviation, and results showed that there were 15 factors with a characteristic root greater than 1. The interpretation rate of the first factor was 23.51%, far below the critical standard of 40%, indicating that there was no serious common method deviation.

### Correlation analysis of self-efficacy, resilience, perseverance, and special situation handling capability

A correlation analysis was performed to assess the relationships between self-efficacy, resilience, perseverance, and special situation handling capability (average scores were used in the analysis). The results are summarized in Table 1 and show that self-efficacy is significantly positively correlated with resilience, but not with special situation handling capability or perseverance; resilience is significantly positively correlated with special situation handling and perseverance; while special situation handling capability is not significantly correlated with perseverance.

**Table 1:** Descriptive statistics and correlation matrix of special situation handling capability and psychological traits.

	M	SD	Flight duration	Self-efficacy	Resilience	Special situation handling capability
Flight duration	175.810	104.400				
Self-efficacy	3.460	0.490	− 0.309**			
Resilience	3.220	0.480	0.115	0.201**		
Special situation handling capability	1.410	0.050	0.211**	− 0.076	0.209**	
Perseverance	2.000	0.210	0.207**	− 0.112	0.214**	0.065

Note: **p < 0.01.

### Relations between self-efficacy and special situation handling capability: a moderated mediation model

#### Main effects test

According to the mediation and moderation test process of Hayes, Wen and Ye’s PROCESS Model 4 was first used to test the mediating effect of resilience between self-efficacy and special situation handling. According to their results, self-efficacy did not significantly predict special situation handling capability after controlling for flight duration (*β* = − 0.01, *SE* = 0.01, *p* = 0.870).

#### Mediating effects test

Self-efficacy and resilience were simultaneously incorporated into a regression equation. Results indicated that self-efficacy did not significantly predict special situation handling capability (*β* = − 0.06, *SE* = 0.01, *p* = 0.360), while resilience significantly predicted special situation handling capability (*β* = 0.20, *SE* = 0.01, *p* = 0.003). The Bootstrap percentile method for offset correction indicated that the mediating effect of resilience on self-efficacy and special situation handling was significant (Indirect Effect = 0.05, Boot*SE* = 0.02); the 95% confidence interval was [0.02, 0.10], excluding 0. Therefore, the mediating effect of resilience on self-efficacy and special situation handling is tenable.

#### Moderating effects test

The PROCESS Model 7 was adopted to test the potentially moderating effect of perseverance on the pilots’ ability to handle special situations. Before analysis, all variables were standardized. The results which are summarized in Table 2, show that the interaction between perseverance and self-efficacy significantly predicted resilience (*β* = 0.14, *SE* = 0.06, *p* = 0.030), indicating that perseverance can moderate the relationship between self-efficacy and resilience.

**Table 2:** Test of moderated mediating effect of self-efficacy on special situation handling capability.

Variable	Equation 1 (criterion: special situation handling capability)			Equation 2 (criterion: resilience)			Equation 3 (criterion: special situation handling capability)		
	*β*	*SE*	*t*	*β*	*SE*	*t*	*β*	*SE*	*t*
Self-efficacy	− 0.08	0.07	− 1.13	0.22***	0.06	3.28	− 0.12	0.07	− 1.84
Resilience							0.23**	0.07	3.51
Perseverance				0.23**	0.06	3.64			
Perseverance * Self-efficacy				0.14**	0.06	2.19			
*R*^*2*^	0.01			0.12			0.06		
*F*	1.27			9.62***			6.82***		

Note: ***p < 0.001.

#### Simple slopes test

**Figure 1:**
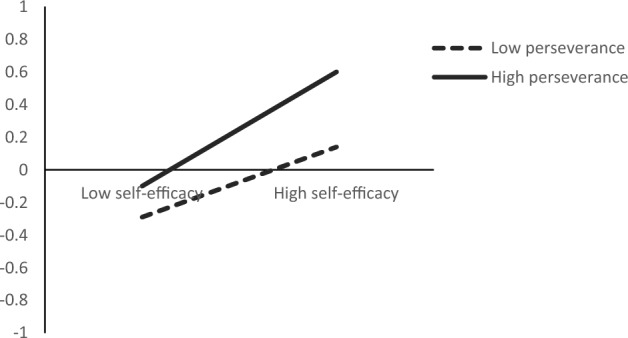
Moderating effect of perseverance.

To explain the essence of the interaction between self-efficacy and perseverance more clearly, self-efficacy and perseverance were divided into high (respondents with scores > 1SD above the mean) and low (respondents with scores < 1SD below the mean) groups. Results showed that self-efficacy did not predict resilience in cadets with low perseverance (*β* = 0.07, *t* = 0.77, *p* = 0.44), while self-efficacy significantly predicted resilience in a positive manner for those with high perseverance (*β* = 0.035, *t* = 4.13, *p* < 0.001)—see Fig. 1.

To sum up, the influence of self-efficacy on special situation handling capability through resilience is moderated by perseverance. Results failed to support the effect of self-efficacy on special situation handling capability through resilience for cadets with low perseverance (Index = 0.02, Boot*SE* = 0.02, 95%CI [− 0.03, 0.07]). However, the effect of self-efficacy on special situation handling capability through resilience was found to be tenable for cadets with high perseverance (Indirect Effect = 0.08, Boot*SE* = 0.03, 95%CI [0.03, 0.16]).

## Discussion

### Research findings

This study explored the influence of pilots’ self-efficacy, resilience and perseverance on their capability of handling special flight situations. The research results partly support our hypothesis. First, this study explored the positive influence of self-efficacy on special situation handling capability and results showed that self-efficacy did not directly influence the capability of handling special flight situations, after controlling for flight duration. Second, this study introduced resilience into the model and explored the influence of resilience on the relationship between self-efficacy and special flight situation handling capability. It was found that resilience plays a mediating role between self-efficacy and flight special situation handling capability. Finally, this study introduced perseverance into the model, and verified the moderating effect of perseverance on the influence of self-efficacy on resilience. In other words, high perseverance strengthens the positive influence of self-efficacy on resilience and improves an individual’s capability of handling special situations.

### Theoretical contribution

This study, with a focus on flight special situation handling, constructed a model of psychological factors that influence special flight situations from the perspective of psychological theory, and performed verification and analysis. For special flight situations, previous studies have mostly focused on changes in pilots’ mental states after special situations have occurred. By comparison, the current study comprehensively judged pilots’ capability of handling special situations, beginning with scenarios of special flight situation handling, and subsequently introducing psychological factors to explore the influence of the combination of those factors on flight safety.

This study enriches the research on the effects of flight psychology. In comparison with previous literature with experience summary and analysis, this study makes a quantitative analysis of pilots’ capability of handling special situations and introduces individual psychological factors into the model of factors that influence pilot’s special flight situation handling capability. It not only investigates the influence of self-efficacy on pilot’s special flight situation handling capability, but also comprehensively explores the interaction of resilience, perseverance and self-efficacy on their special situation handling capability. The results and conclusions have deepened our understanding of the mechanisms underpinning pilots’ special situation handling.

### Limitations

First, this study explored the mediating mechanism of self-efficacy and the mediating effect of perseverance on special flight situation handling capability. However, there may be other factors that could be incorporated into the model, for example, safety motivation and other cognitive factors might also influence the relationship. However, previous research has shown that safety motivation can significantly influence an individual’s performance level^25,26^. Therefore, in future studies, these variables could be incorporated into the model, to further investigate the influence of different variables on the capability of handling special flight situations and construct more accurate theoretical models for special situation handling capability.

Second, the samples in this study were selected from a flight brigade in Northeast China and whether the research findings are applicable to military pilots or civil aviation pilots in other regions requires further evaluation. In future studies, the number of military pilot samples might be expanded, civil aviation pilots’ handling capability, psychological quality and other information could be incorporated into the model, and cross-level analysis of pilots of different aircraft types (e.g. fighter, bomber, early warning aircraft, transport aircraft, tanker, and helicopter) conducted, to improve the accuracy and universality of the model.

Finally, this study used cross-sectional methods to collect and analyze data and lacked longitudinal data which will allow causality to be assessed. As a result, there may be some measurement errors that affect the model’s results. Moreover, the data were obtained from self-rating scales, which may lead to some common method deviations. In further studies, we will combine computer science and virtual reality technology, and use the scenario judgment test with higher degrees of simulation to judge pilots’ special situation handling capability. The model will also be improved and optimized by designing accurate and rigorous longitudinal studies to improve the special situation handling capability of pilots, ensure China’s aviation safety, and provide psychological theoretical support and technical support for intelligent air combat.

## Conclusions

This paper aims to examine the psychological factors that influence pilots’ ability to handle special situations by constructing a psychological path model. Specifically, this study explores the mediating role of psychological resilience in the relationship between self-efficacy and pilots’ ability to handle special situations, as well as the mediating role of perseverance. (More specifically, the results indicate that self-efficacy positively influences psychological resilience, which, in turn, leads to better handling of special situations.) Through an in-depth analysis of survey data, the study found that self-efficacy, through the intermediary variables of psychological resilience, can affect the military pilots’ ability to handle special situations to some extent. Therefore, it is recommended to include psychological resilience training during flight aviation school’s special handling training to enhance the training’s effectiveness. Moreover, this study concludes that the psychological structure that affects the handling ability of special flight circumstances is complex and suggests that future research should consider more factors to refine the psychological influencing factor model of handling special flight situations. This conclusion provides theoretical underpinnings and practical support for improving pilots’ handling ability.

## Data Availability

Due to the confidentiality requirements of pilot information, the data set generated during the current research period is not publicly available, but it can be obtained from the corresponding author through email inquiry.
